# GIS habitat analysis for lesser prairie-chickens in southeastern New Mexico

**DOI:** 10.1186/1472-6785-6-18

**Published:** 2006-12-04

**Authors:** Kristine Johnson, Teri B Neville, Paul Neville

**Affiliations:** 1 Natural Heritage New Mexico, University of New Mexico, Biology Department, MSC03 2020, 167 Castetter Hall, Albuquerque, NM 87131, USA; 2 Earth Data Analysis Center, University of New Mexico, MSC01 1110, 111 Bandelier West, Albuquerque, NM 87131, USA

## Abstract

**Background:**

We conducted Geographic Information System (GIS) habitat analyses for lesser prairie-chicken (LPCH, *Tympanuchus pallidicinctus*) conservation planning. The 876,799 ha study area included most of the occupied habitat for the LPCH in New Mexico. The objectives were to identify and quantify: 1. suitable LPCH habitat in New Mexico, 2. conversion of native habitats, 3. potential for habitat restoration, and 4. unsuitable habitat available for oil and gas activities.

**Results:**

We found 16% of suitable habitat (6% of the study area) distributed in 13 patches of at least 3,200 ha and 11% of suitable habitat (4% of the study area) distributed in four patches over 7,238 ha. The area converted from native vegetation types comprised 17% of the study area. Ninety-five percent of agricultural conversion occurred on private lands in the northeastern corner of the study area. Most known herbicide-related conversions (82%) occurred in rangelands in the western part of the study area, on lands managed primarily by the US Bureau of Land Management (BLM). We identified 88,190 ha (10% of the study area) of habitats with reasonable restoration potential. Sixty-two percent of the primary population area (PPA) contained occupied, suitable, or potentially suitable habitat, leaving 38% that could be considered for oil and gas development.

**Conclusion:**

Although suitable LPCH habitat appears at first glance to be abundant in southeastern New Mexico, only a fraction of apparently suitable vegetation types constitute quality habitat. However, we identified habitat patches that could be restored through mesquite control or shin-oak reintroduction. The analysis also identified areas of unsuitable habitat with low restoration potential that could be targeted for oil and gas exploration, in lieu of occupied, high-quality habitats. Used in combination with GIS analysis and current LPCH population data, the habitat map represents a powerful conservation and management tool.

## Background

### LPCH population and habitat declines

The LPCH is a prairie grouse found in upland shrubland and grassland habitats of the Great Plains. Except for the Gunnison's sage-grouse (*Centrocercus minimus*), the LPCH has the most restricted distribution and smallest population size of any native North American grouse species. Distribution has declined by over 90% since the 1800s [1]. Significant reductions in population size and distribution during that time have been attributed to excessive grazing of rangelands, conversion of native rangelands to croplands, drought, and chemical control of sand sagebrush (*Artemesia filifolia*) and shin-oak (*Quercus havardii*). As a consequence, populations are now fragmented across its range [1].

### Status and conservation efforts

The US Fish and Wildlife Service classifies the LPCH as a candidate for protection under the Endangered Species Act. In January 2003, representatives of state and federal agencies, ranchers, the oil and gas industry, and conservation organizations formed a working group to create a conservation strategy for two candidate species, the LPCH and sand dune lizard (SDL, *Sceloporus arenicolous*) in shinnery oak and sandsage grassland communities in New Mexico. We follow terminology in Peterson and Boyd [2], where the term "sand shinnery" refers to the dunal vegetation community dominated by "shin-oak" shrubs, *Quercus havardii*.

The working group participated in a series of strategic planning meetings over a two-year period and produced a planning document in August 2005 [3]. For the creation and implementation of the strategy, it was essential to understand the distribution of occupied, suitable, restorable, and unsuitable LPCH habitat. The working group requested some habitat analyses for conservation planning; others grew from our questions and ideas. Here we report on the habitat analyses we performed and their implications for LPCH conservation.

### Habitat analysis needs and objectives

**Figure 1:**
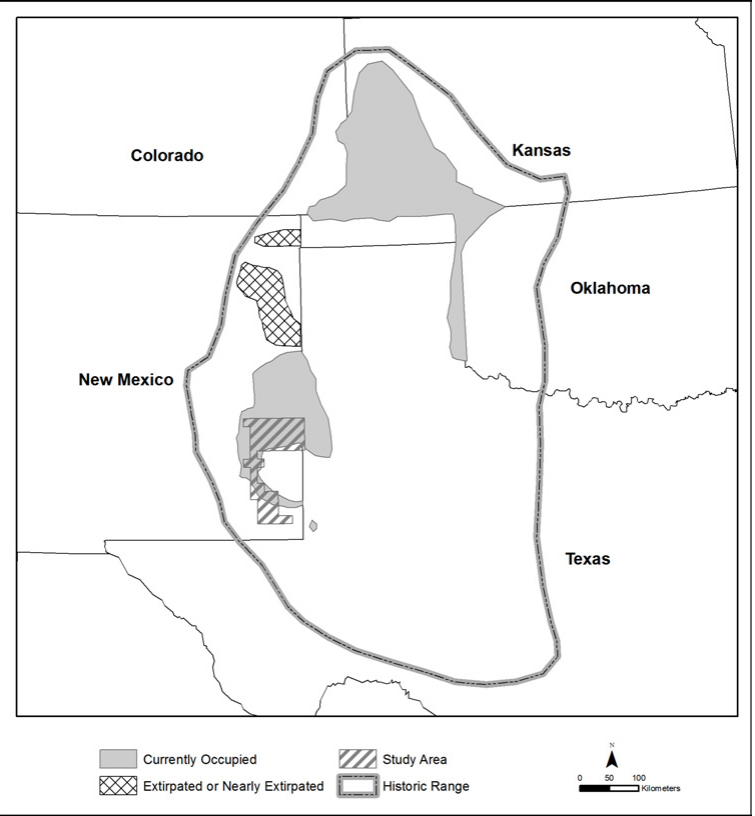
**Current and historical ranges of the lesser prairie-chicken**. Ranges adapted from [1, 39].

The LPCH occurs in habitats dominated by shin-oak or sand sagebrush with tall grass or mixed-grass species, in five states within the Southern Shortgrass Prairie Ecoregion [4,5]: southeastern Colorado, southwestern Kansas, western Oklahoma, eastern New Mexico, and the Texas Panhandle and portions of Texas contiguous with the New Mexico range (Figure 1). This study focuses on occupied habitats in New Mexico.

LPCH typically nest on the ground under sand sagebrush or shin-oak shrubs, or in residual bunchgrasses (e.g., *Aristida *spp.*, Schizachyrium *spp.*, Andropogon *spp.) [1,6]. Predation is the primary cause of nest failure. Nest depredation and abandonment rates and adult survivorship have been associated with vegetation height, cover, and density [7,8]. Although residual tall grass appears important for nesting cover, LPCH hens prefer to nest in pastures containing a mixture of grass and shin-oak over pastures in which shrubs have been eliminated [9,10]. LPCH in New Mexico prefer shin-oak for brood rearing, due to the cover, acorns, and abundant insects it provides [11]. Although limited use of herbicides is sometimes recommended to achieve a balance of shin-oak and grass, large-scale conversion of shin-oak/grass communities to grasslands is considered detrimental [3,9-11].

The minimum habitat patch size required to support LPCH is unclear, but research has focused on two patch sizes: ~3,200 ha and ~7,200 ha (representing areas with approximately two and three mile radii, respectively, around a point, typically a lek site). Taylor and Guthery [12] proposed an area of suitable habitat with radius of 3.2 km centered around a lek (equivalent to 32 km^2^, 3,200 ha, or a 2 mi radius) as the minimum patch size for maintenance of a "lek population," because 90% of activity occurred within this limit. This distance was consistent with earlier studies [13,10]. Giesen [6] found that the mean distance from lek of capture to nest site was 1.8 km (range 0.2–4.8 km). Although Pitman et al. [14] found some nests located further from the capture lek, for most years mean distances were consistent with those listed above. Thus, a 3.2 km radius can be considered an estimate of the minimum breeding-season patch size around a lek site needed by the majority of LPCH hens attending that lek.

In addition, Taylor and Guthery [12] proposed 72 km^2 ^as the optimum patch size needed for maintaining healthy LPCH populations, based on the observation that virtually all detections of LPCH were within 4.8 km (3 mi) of the display ground. This observation was corroborated by Giesen [6]. The 3 mi/7,200 ha patch size was subsequently used by Woodward, Fuhlendorf and co-authors, who found that landscapes in which LPCH populations declined were characterized by greater rates of landscape change and greater rates of shrub loss within 4.8 km of leks than landscapes in which populations did not decline [15]. An investigation of the scale-dependent effects of habitat loss and fragmentation on LPCH populations found that general landscape changes, amount of cropland, and number of trees impacted LPCH populations at the 4.8 km (7238 ha) scale [16].

Both of these frequently cited patch sizes have biological significance, but both are based on distances smaller than observed dispersal distances [[1] and references therein]. Thus, they represent only minimum requirements, and conservation planning based on even the 7,200 ha patch size will not guarantee LPCH population stability. The analyses we performed using these two patch sizes should therefore be considered useful primarily to identify areas where minimum-sized patches of suitable LPCH habitat remain.

Fragmentation and conversion of sandhill landscapes have occurred throughout eastern New Mexico. Mechanisms that fragment the landscape are scale-dependent [16] and differentially impact LPCH populations at local and regional scales. In one study, regional scale (7,238 ha) landscape impacts included conversions to cropland and tree encroachment. Changes in edge density and largest patch size were important variables at small spatial scales (452–1,800 ha) [16]. Braun et al. [17] suggested that the short dispersal distances and specialized food habits of grouse may make them relatively intolerant of extensive habitat fragmentation.

To manage for conservation of LPCH, it is necessary to understand the abundance and distribution of suitable habitat on the scales at which management action occurs. For example, individual ranchers participating in habitat conservation agreements and NM Department of Game biologists managing Fish Prairie-Chicken Conservation Areas operate locally. Regional managers include federal agencies such as the BLM. The purpose of this project was to perform GIS analyses of LPCH habitat in the study area. Objectives were to identify and quantify: 1. suitable habitat, 2. conversion of native habitats, 3. potential for habitat restoration, and 4. unsuitable habitat available for oil and gas activities.

## Results

### Land conversion

**Figure 2:**
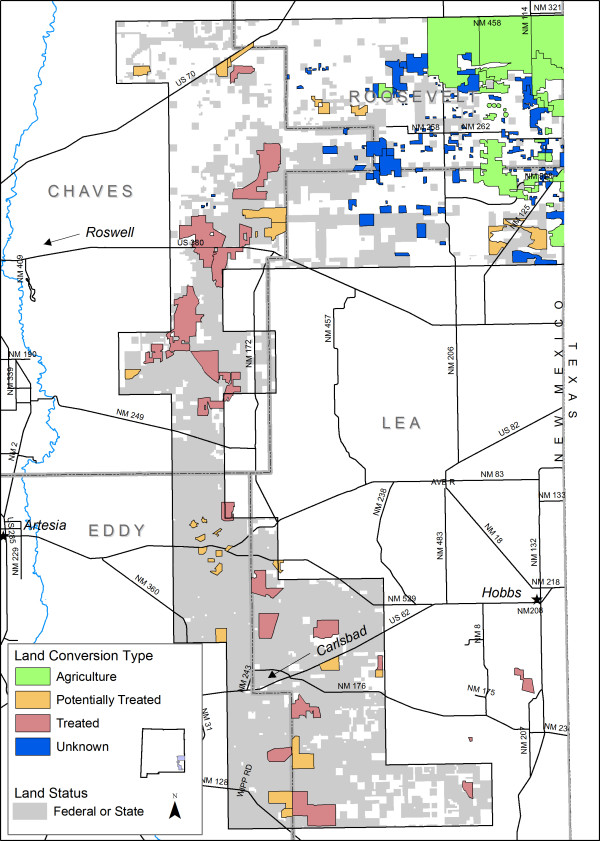
**Land cover conversions in the study area**. Treated = treated with herbicides.

To identify herbicide-treated areas, we used the Normalized Differential Vegetation Index (NDVI) image in conjunction with BLM environmental assessment decision documents indicating the boundaries of areas treated with herbicide from 1981 to 1993 (see Methods, below). The land conversion analysis revealed that 44,021 ha (5% of the study area) were treated with herbicides, which converted shrubland to grassland vegetation types. Based on the NDVI, we classified 19,071 (2%) additional hectares as potentially treated. We classified 56,193 ha (6%) as converted to agriculture, and 27,119 ha (3%) as unknown conversion (Figure 2). The total area apparently converted from native vegetation types was 146,405 ha, 17% of the study area.

**Figure 3:**
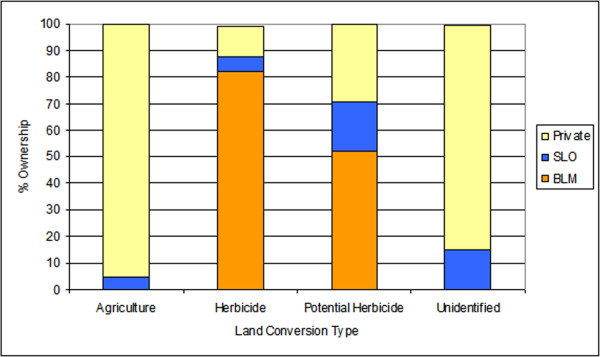
**Land cover conversion types by land ownership**. SLO=State Land Office, BLM= US Bureau of Land Management.

Considering all categories of land conversions, private lands accounted for 60% and BLM lands 32%. The majority of agricultural conversions occurred on private lands, while most herbicide treatments occurred on BLM rangelands, (Figure 3). Potentially treated areas were also found primarily on BLM lands, with smaller areas on private and state land. Unknown conversions occurred primarily on private land (Figure 3). Based on known land use nearby, these unknown areas probably represent mainly agricultural conversion.

### Habitat suitability and patch size

We found 355,515 ha of Group A habitats, suitable habitats dominated by shin-oak or sand sagebrush (see Methods, Habitat Analysis, for a definition of habitat suitability), covering 41% of the study area. Habitats defined as seasonal use or transitional areas (Group B) covered 44,186 ha (5% of the study area) and included mixed grasses with some shin oak (Table 1). Most of the potential restoration habitat (Group C, 44,006 ha, 5% of study area) occurred in the south, and unsuitable habitat (49% of study area) occurred throughout the study area.

**Table 1:** Vegetation map units, areas, and groupings according to LPCH habitat suitability.

MU#	Group	MU Description	Hectares	Group Totals
1	A	Shin-Oak/Mixed Mid-Grass & Tall-Grass Duneland	82642	
2	A	Shin-Oak/Sparse Duneland	73894	
3	A	Shin-Oak/Mixed Mid-Grass & Tall-Grass Shrubland	26232	
5	A	Shin-Oak/Mixed Mid-Grass & Short Grass Shrubland	139591	
6	A	Shin-Oak/Sparse Shrubland	21081	
15	A	Shin-Oak-Sand Sagebrush Shrubland	2921	
8	A	Sand Sagebrush Shrubland	9153	
		*Total Group A habitat*		***355515***
24	B	Mixed Grasses/Shin-Oak Grassland	23465	
23	B	Mid-Grass Grassland	17868	
13	B	Tall-Grass Grassland	2854	
		*Total Group B habitat*		***44187***
		*Total lesser prairie-chicken habitat*	***399702***	
7	C	Honey Mesquite-Shin-Oak/Short-Grass Shrubland	35626	
10		Honey Mesquite Shrubland	55321	
14		Honey Mesquite Sparse Shrubland	55521	
11		Escarpment-Footslope Shrubland	2515	
		*Total non shin-oak shrubland*	***148983***	
		*Total shrubland*	***504498***	
16		Short-Grass Grassland	145906	
25		Short-Grass/Honey Mesquite Grassland	61259	
28	C	Treated Mixed Mid-Grass and Tall-Grass Grassland	8379	
		*Total Group C*		***44006***
		*Total grassland*	***259731***	
19		CRP Fields	13482	
20		Agricultural Fields	19198	
26		Playa Lakebed	2278	
27		Barren/Sparsely Vegetated/Manmade Disturbance	77612	
		*Total Other*	***112570***	
		*Study area total*	***876799***	

Group A – occupied and suitable, Group B – seasonal and transitional use, Group C – potential restoration areas. Areas calculated using ERDAS Imagine.

**Figure 4:**
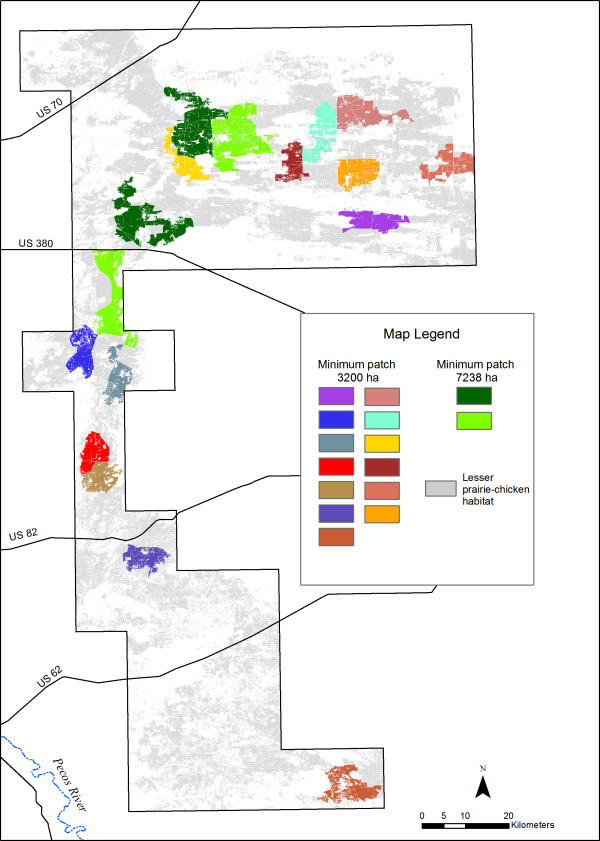
**Minimum patch sizes of LPCH habitat in the study area**. LPCH habitat is defined as shin-oak dominated (Group A) mapping units.

The analysis revealed 55,167 ha of Group A Map Units (MUs, 16% of suitable habitat and 6% of the study area) distributed in 13 patches of at least 3,200 ha (3,200–7,237 ha in area, Figure 4). We found 38,033 ha (11% of suitable habitat and 4% of the study area) distributed in only four patches over 7,238 ha. The largest patches were contained in two areas, one of which is south of US 380 in the sparse and scattered population area [3] and outside the PPA designated by the working group. All other patches of Group A MUs were smaller than 3,200 ha, comprising a total of 262,315 ha. Thus, 74% of "suitable" LPCH habitat occurred in patches too small to support LPCH, as defined by current literature, leaving only 26% of suitable habitat and 11% of the study area suitable for LPCH. Patches of at least 3,200 ha occurred primarily on private land (45%), followed by BLM (36%) and state (20%) land. Patches of over 7,238 ha also occurred primarily on private land (39%), followed by BLM (37%), and state (25%) land.

**Figure 5:**
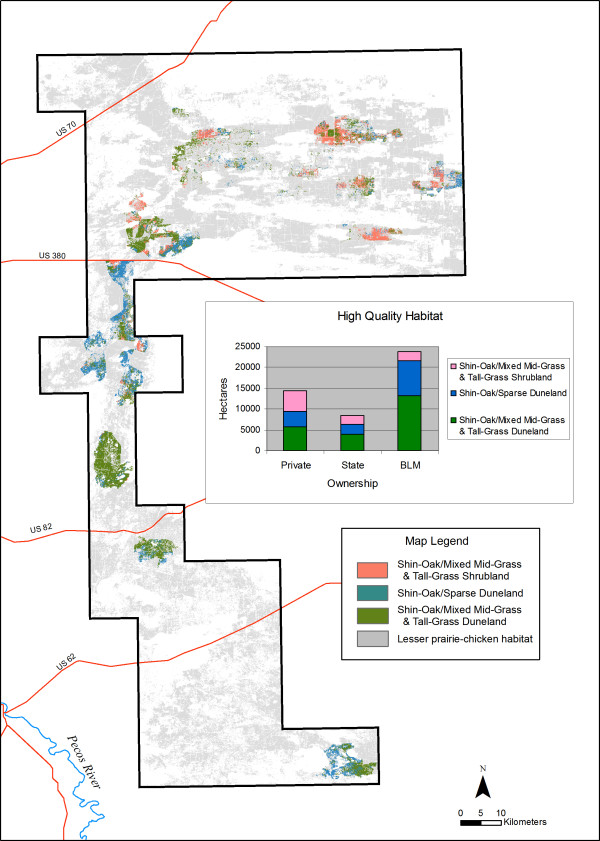
**High-quality LPCH habitat in moderately large patch sizes**. High-quality habitats are defined as MUs 1, 2, and 3. Patch size is 3,200 ha or larger.

The analysis revealed 46,558 ha (5% of the study area) of the highest-quality habitat (MUs 1, 2, and 3; Table 1) distributed in patches of 3,200 ha or larger (Figure 5). BLM manages the majority of these large, high-quality habitat patches (51%), followed by private landowners (31%) and the state (18%). Although the high-quality patches are smaller in size than the optimal patch size of 7,200 ha suggested by several authors [6,12,15,16], they offer opportunity for habitat improvement through expansion of patch size. We therefore chose to use the smaller patch size in our analysis, to include substantial areas of moderately sized patches of the best LPCH habitat.

### Restoration habitat

**Figure 6:**
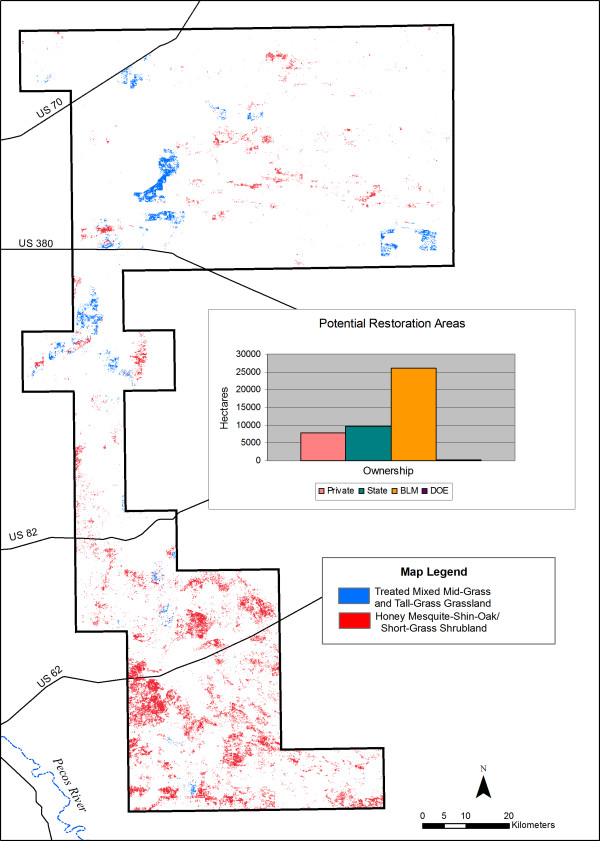
**Potential LPCH habitat restoration areas**. Inset shows restoration potential by landowner. Restoration habitats contain altered vegetation types that were originally LPCH habitat and areas that have been impacted by invasive species but still contain enough suitable vegetation to allow successful restoration. DOE = Department of Energy.

We identified 44,006 ha (5% of the study area) of habitats with reasonable restoration potential (Figure 6). Habitats with the highest restoration potential occur primarily in the southern portion of the study area, with little restoration habitat in the central portion. The largest share of restoration habitats occurs on BLM land (59%). The restoration habitats on BLM lands in the south are largely due to honey mesquite invasions into shin-oak habitats, while restoration areas in the north would require re-introduction of shin-oak into areas where it has been destroyed. Eighty-one percent of potential restoration areas would require removal of honey mesquite in shin-oak habitats.

### PPA analysis/oil and gas development

**Figure 7:**
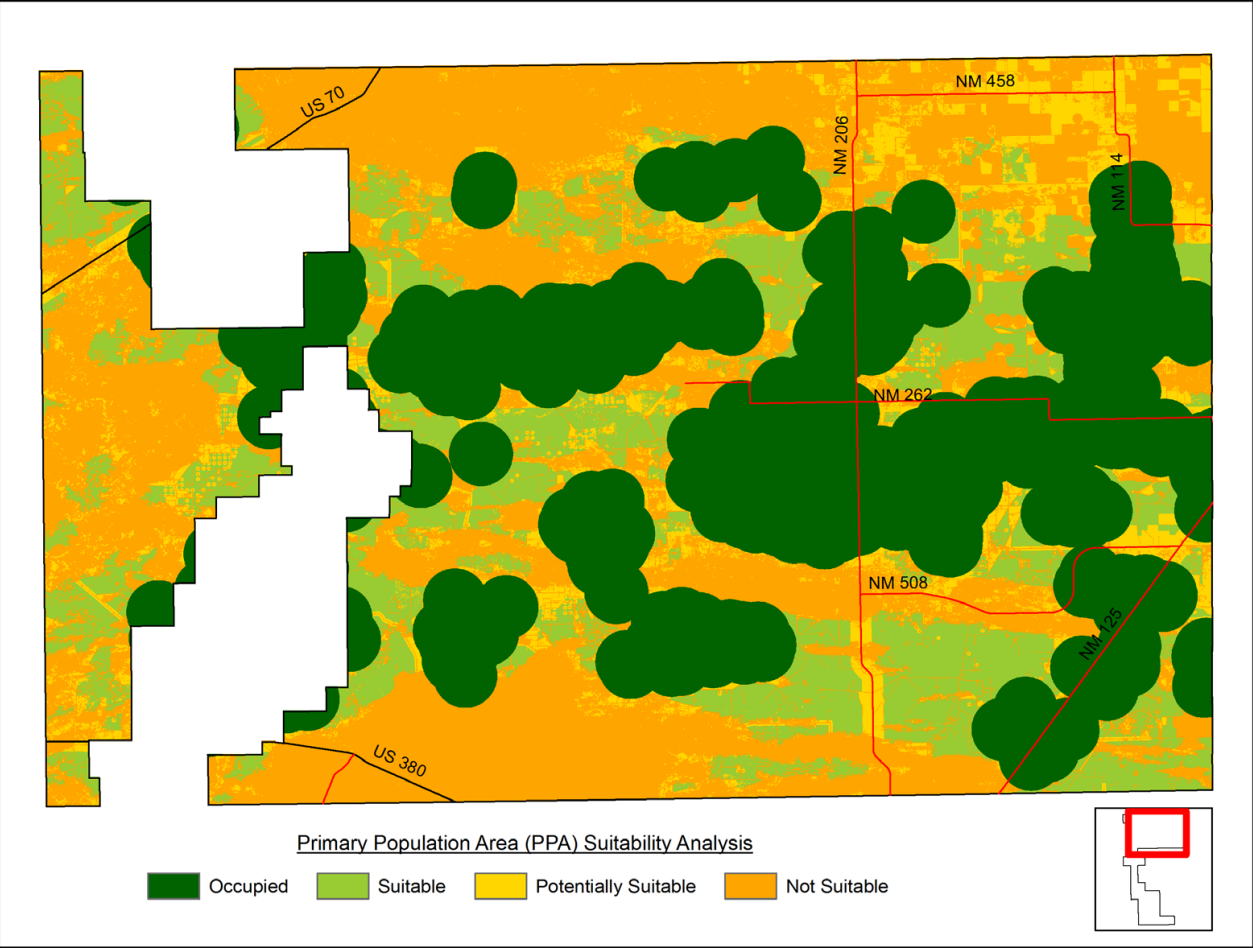
**Habitat management categories within the primary population area**. Occupied habitat – all areas within 2.4 km (1.5 mi) of an active LPCH lek site, regardless of vegetation. Suitable habitat – unoccupied areas of appropriate vegetation type, in patches of 129.5 ha (320 ac) or more, falling entirely outside of Robel [25] impact/avoidance distances around infrastructure. Potentially suitable habitat – unoccupied areas of appropriate vegetation type in patches of less than 129.5 ha and/or falling within Robel impact/avoidance distances around infrastructure. Unsuitable habitat – areas outside of appropriate vegetation, including urban and agricultural areas, areas where shin-oak is naturally not present or has been eliminated by chemical treatment, or other areas where natural vegetation has been greatly altered or degraded.

We performed separate GIS analyses to map occupied, suitable, potentially suitable, and unsuitable habitat types within the PPA and to determine how conservation actions might impact the oil and gas activities (See definitions in Methods, below; Figure 7). The PPA is a 424,522 ha area defined by the working group in N Lea, S Roosevelt, and NE Chaves Counties that contains the highest concentrations of LPCH in NM. The analysis revealed 162,729 ha of occupied habitat (38% of the PPA) and 68,787 ha of suitable habitat (16% of the PPA). Within the PPA, 31,680 ha (7%) were deemed potentially suitable and 161,351 ha (38%) were unsuitable LPCH habitat. Using this analysis, industry representatives were able to evaluate proposed strategies for restrictions on new mineral leasing on the PPA.

### Future analyses

GIS habitat analyses will be instrumental to implementing conservation actions such as mesquite reduction or eradication on rangelands or limited use of herbicides to achieve vegetation standards for quality habitat. Herbicide use is recommended only in small blocks, away from dune areas, and over 1.5 miles away from active lek sites. Restoration priorities must consider connectivity, proximity, and patch size. Ongoing GIS analyses will be necessary to identify sites that meet these and other recommendations of the conservation plan.

## Discussion

### Map units

**Figure 8:**
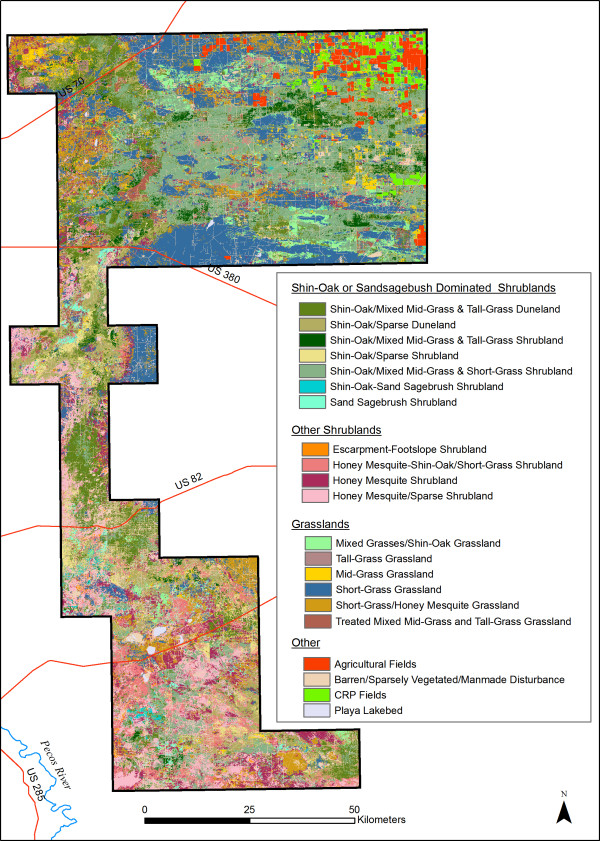
**LPCH habitat map**. For distinction between habitat map and vegetation map, see P. 10, Habitat map.

The map has twenty-one MUs (Table 1, Figure 8). Because of the focus on LPCH habitat needs, some mapping units (MUs) appear "lumped" and others "split," relative to more standard vegetation classification systems. For example, MU 1, Shin-Oak-Mixed Mid-Grass and Tall-Grass Duneland, and MU 3, Shin-Oak- Mixed Mid-Grass and Tall-Grass Shrubland, have similar species composition. The primary difference between these two MUs is topographical rather than vegetative. We define these separately for several reasons. First, SDLs occur in dunes [18]; thus, differentiation of dune areas is potentially useful for SDL management. Second, LPCH have been reported to preferentially nest in dunes [1]. Finally, several previous vegetation classifications of the sand shinnery community have differentiated dunes from areas lacking dunes [[2] and references therein, pp. 3–4].

The map also differentiates habitats that differ in overall vegetation cover and shrub versus grass density. Nest success has been shown to correlate with height, density, and abundance of residual grasses, especially bluestem (*Andropogon *spp*. *and* Schizachyrium *spp.), near nests [7,19]. Brood foraging sites have been described as having taller shrubs, greater canopy cover, and greater shrub density than surrounding areas [6,20,21]. Thus, it is useful to differentiate habitats that differ in overall vegetation cover and shrub versus grass density. MUs designated as "sparse" (e.g., Shin-Oak/Sparse Duneland and Shin-Oak/Sparse Shrubland) contain lower grass cover than corresponding MUs not so designated.

In contrast, MUs 16, 23, and 13 (Short-Grass, Mid-Grass, and Tall-Grass Grassland, respectively) each contain several grassland associations that might be grouped differently using a more traditional vegetation mapping methodology such as the International Terrestrial Ecological Systems Classification [22], a mid-scale classification system, or the hierarchical, community-scale approach of the US National Vegetation Classification [23]. Because none of the plant associations individually contained in MUs 16, 23, or 13 constitutes preferred habitat for LPCH, the map combines structurally similar but compositionally different grassland associations. Structural components rather than affinity to specific species dictate the use of these grasslands by LPCH.

### Land conversion

Pasture-scale treatment with the herbicide tebuthiuron occurred on public lands in the study area from the 1980s until the early 1990s and is still occurring on private lands. Treated areas contain substantially lower shin-oak cover than untreated areas and were therefore relevant to the mapping effort [10]. Twenty years after treatment, the NDVI showed that areas known (from BLM Roswell, NM Field Office records) to be treated still differed markedly in shrub composition from untreated areas. Persistence of the effects of treatment provides further rationale for distinguishing shrub-dominated from grass-dominated habitats.

The effects of treatment varied with timing of treatment, quantity of herbicide used, and subsequent management practices. Thus, identifiable digital signatures representative of all treatment areas were not evident on the imagery, and treatment areas were not readily seen in the initial map. We therefore created a separate layer of the treatment areas (Figure 2). Over half of the existing Tall-Grass Grassland (54%) and 47% of the Mid-Grass Grassland resulted from herbicide treatments. Our analyses demonstrate that 17% of the study area has been converted by human activities from native vegetation types into agricultural fields, shrub-free grasslands, or other types of disturbance. Only 5% of the study site shows good potential for restoration. This layer will be useful for analyses of the effects of herbicide treatment on habitats of LPCH, SDL, and other wildlife.

### Habitat suitability, patch size, and restoration

Within the mapped area, only three areas contain large patches of suitable habitat, and one of those is south of US Highway 380, where LPCH populations are sparse and scattered (Figure 4). The GIS analyses also indicate that most high-quality habitat occurs in patches smaller than 3,200 ha (Figure 5), rendering them by most definitions below the minimum size required by LPCH.

The presence of infrastructure that either kills or at least deters LPCH arguably alters habitat quality [24,25], but it is not immediately obvious if and how patch size requirements are expected to vary with infrastructure density. Our analyses are concerned with minimum patch sizes of suitable habitat; increasing infrastructure density would change suitability. Because LPCH are known to avoid infrastructure [25], we would expect them to move longer distances in habitat with more infrastructure, in an attempt to find infrastructure-free patches. This is apparently the case in Oklahoma [24]. At some point, however, it would become unprofitable to disperse further and birds should settle for some infrastructure, which they appear to do in Oklahoma, possibly altering life history patterns to compensate for the impacts of the infrastructure on survivorship.

Honey mesquite has been invading parts of the Southwest for decades, likely due to livestock grazing practices [26-28]. Honey mesquite MUs would require major restoration efforts focused on honey mesquite removal. In contrast, Group C restoration habitats in the north (in the PPA) include primarily Honey Mesquite-Shin-Oak/Short Grass Shrubland and Treated Mixed Mid-Grass and Tall-Grass Grassland, any of which should be easier to restore to suitable vegetation types through shin-oak introduction.

### PPA analysis/oil and gas development

Nesting and non-nesting LPCH have been shown to avoid structures associated with oil and gas activity, such as wellheads, roads, and electric transmission lines [25]. The working group was therefore interested in excluding oil and gas activities from suitable and restorable LPCH habitat. Excluding the areas of occupied, suitable, and potentially suitable habitats available for LPCH in the PPA, the GIS analyses revealed at least 161,351 ha of unsuitable habitat with low restoration potential, where the working group could consider allowing oil and gas activities to occur. The potential for identifying areas where human impacts on sensitive species habitats will be lowest is one of the most useful contributions of these analyses.

Note, however, that the amount of "suitable" and "unsuitable" habitat depends on how those categories are defined. The habitat quality analysis for the entire study area included only large patches of the most suitable MUs and, as a result, it identified only 46,558 ha of high-quality habitat in the entire study area (5% of the entire area). Because so few large habitat patches exist, to increase the area of suitable habitat, smaller and lower-quality habitat patches would need to be protected and restored, especially in the PPA where the majority of LPCH occur. The working group determined that management should be based on a more liberal definition of LPCH habitat. For the PPA/oil and gas analysis, LPCH habitat was defined to include more vegetation types, smaller patch sizes, and unsuitable vegetation types near active leks. As a result, the PPA analysis performed for conservation planning identified 263,196 ha (62% of the much smaller PPA) as occupied, suitable, or potentially suitable habitats where oil and gas development should be excluded.

## Conclusion

A detailed habitat map at a scale of 1:24,000, together with GIS analyses requested by the Lesser Prairie-Chicken/Sand Dune Lizard Working Group, provided: 1. insight into the area, distribution, and quality of available habitat for the LPCH, 2. information for stakeholder negotiations, and 3. the basis for creation and implementation of a conservation strategy for the two species.

Although suitable LPCH habitat appears at first glance to be abundant in southeastern New Mexico, consideration of plant community type and patch size revealed that only 26% of the study area was covered in large patches of suitable vegetation types. Given the scarcity of habitats with potential to contribute to the species' recovery, it is important to focus the inevitable human impacts in areas of unsuitable habitat and to restore degraded vegetation and small patches of suitable vegetation types. Restoration efforts could be focused in two habitat types. In the north, shin-oak could be re-introduced into herbicide-treated areas. In the south, mesquite could be removed from areas where it has encroached.

Used in combination with GIS analysis and current LPCH population data, the map represents a powerful management tool. Having participated in the working group, we find it difficult to imagine how such a stakeholder process could be productive without reliance on similar habitat analyses.

## Methods

### Study area

**Figure 9:**
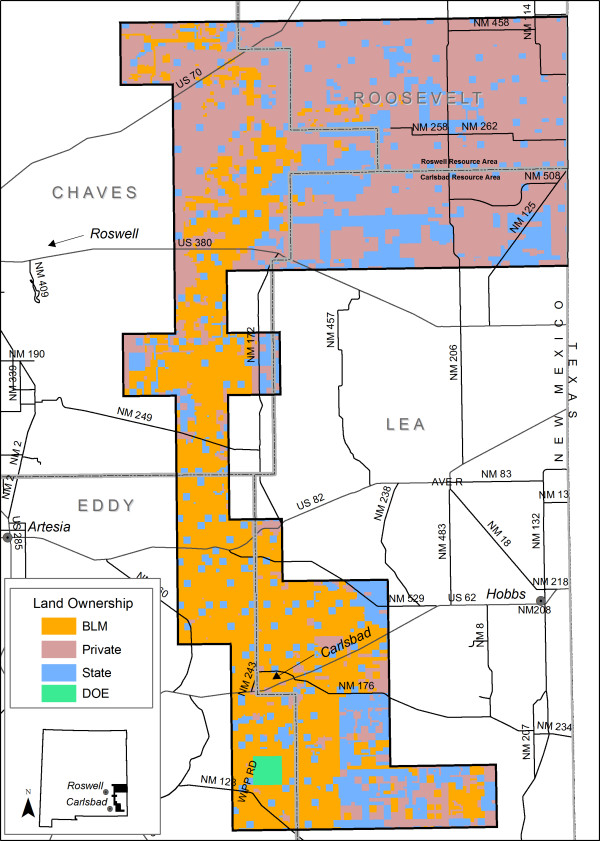
**Study area**. Study area showing land ownership.

The study area comprises fifty-six 7.5' quadrangles, approximately 876,799 ha in portions of Chavez, Roosevelt, Eddy, and Lea counties in southeastern New Mexico (Figure 9). The western edge of this region receives about 330 mm (Roswell) of annual precipitation, with progressively more moisture eastward to the state line (450 mm, Clovis), but less to the south (300 mm, Carlsbad). Most precipitation comes from convective thundershowers during the summer [29], and snow can occur from October to April. Temperatures range from -22.8°C to 45.6°C. The distribution and depth of the sandsheets and underlying calcium carbonate-rich soils determine the growth, density, and distribution of the dominant plant, shin-oak, within the study area. Sand shinnery communities are some of the least understood and most poorly described communities in the southwestern United States [30].

### Habitat map

We mapped the major LPCH habitat types in a large portion of the current range in New Mexico (Figure 1). Our approach to mapping habitat was to: (1) define suitable LPCH habitat by reviewing published literature, consulting experts, and incorporating occurrence data; (2) identify variation in vegetation communities using satellite imagery and aerial photos, (3) collect abundance, floristic, and habitat suitability data on vegetation plots; (4) create MUs relevant to the needs of the LPCH; and (5) analyze and map LPCH habitat quality using GIS. The map is intended for analysis of LPCH habitat preference and use, habitat management for LPCH and other wildlife species, and monitoring of wildlife habitat condition. As such it is not a traditional vegetation map but instead emphasizes plant associations and landforms known to be important to the LPCH and, to a lesser extent, associated species (Figure 8) [31].

The map has been created iteratively, several 7.5' quadrangles at a time. Ten quads were created in 2001, 40 were added in 2003, six were completed in 2004, and five more are in progress in 2006. As new sections were added, plant associations were either included in existing MUs or new MUs were added. The map currently covers 876,799 ha. The minimum MU size (resolution) is 20 × 20 m, meaning that the MUs were designed to be optimally useful at the 1:24,000 scale. It comprises 21 MUs (Table 1).

We used two types of imagery to create the habitat map, Landsat Enhanced Thematic Mapper^+ ^(ETM^+^) satellite imagery and digital aerial photography, digital orth-ophoto quadrangles (DOQs). ETM^+ ^imagery records unique spectral signatures of spectrally similar plant communities, but it has relatively low resolution (30 m × 30 m). As a complement to ETM^+ ^imagery, DOQs provide a limited spectral profile but contribute 1-m spatial resolution. We used several additional data sets, including GIS layers for roads, land status, and topography. We adopted a supervised classification strategy, for which *a priori *knowledge about an area is used to select pixels within the image as training samples to be used by the computer program to identify pixels with spectrally similar characteristics. Thus, the user "supervises" the classification of pixels to specific classes. For each segment added to the map, we made one to three trips to the field to refine MUs and check draft map accuracy. MU definitions are based on three kinds of information: 1. vegetation assemblages, as in a typical vegetation or land cover mapping process, 2. landscape features characteristic of habitat types required by LPCH, and 3. existing knowledge of LPCH and SDL use of various vegetation and habitat types. Details of image processing and MU classification methods are provided in Neville et al. 2005 [31].

We assessed the accuracy of the first 169,386 ha of the map, 18% of what is now completed. We visited 43 sites on BLM land that had not previously been used in the classification. Each site contained over 500 m^2^ of contiguous cells of the same MU, was sampled in proportion to their occurrence on BLM land in the study area, and was a minimum of 100 m from the nearest road. The polygons to be sampled were plotted on 7.5' quadrangle maps containing no vegetation class information. We collected the following data from at least three 10 m × 10 m plots at each site: percent cover of the dominant shrub, dominant grass, dominant sub-shrub, bare ground, and litter; shrub and grass height; landform; location; and comments. We took a photo and made a preliminary assignment to a MU. The accuracy of the map was 84% (Table 2).

**Table 2:** Error matrix for habitat map.

		MU from Field Check														
		1	2	3	4	5	6	7	8	10	11	12	13	14	15	
MU from Map	1	8	1													9
	2		1			1										2
	3			2			1									3
	4				2											2
	5					3	1									4
	6						1									1
	7							2			1					3
	8								2					1		3
	10									2						2
	11										3					3
	12											4				4
	13								1				1			2
	14													2		2
	15														3	3
		8	2	2	2	4	3	2	3	2	4	4	1	3	3	43

Of 43 assessment plots, 36 were correctly assigned to vegetation community, resulting in 84% accuracy. Reasons for error: assessment polygons fell in mixed communities (2); community was correctly identified, but percent cover was misrepresented (3); plots were incorrectly classified (2).

It is a habitat map, as opposed to a land cover map, because it implies more than vegetation type and structure [32]. MU definitions take into account sand dunes, vegetation density, and human land uses and impacts. MU definitions also incorporate plant associations and topographic features known to be important to the LPCH. Our habitat classification contains six MUs dominated by shin-oak, the dominant shrub species of LPCH habitat in New Mexico [1,2]. Because LPCH use shin-oak dunes and plains differently [[2] and references therein, [33]], we split shin-oak-dominated MUs into those with and without dunes and further divided those based on vegetation cover.

In contrast, some grassland communities were lumped. For purposes of this map, grass species composition is most important in the sand shinnery communities preferred by LPCH but is overall less important than the structure provided by those grasses. Percent composition, grass height, and cover of grasses can vary across the study area, depending on livestock stocking rates and rainfall amounts. We defined grassland MUs accordingly. Detailed descriptions of the MUs are available at the Natural Heritage New Mexico web publication list [31].

### Habitat analyses

#### Habitat suitability and patch size

Although the habitat map is potentially useful for SDL habitat analyses, the analyses reported here focus on LPCH. We aggregated the MUs into four landscape-scale units for application to conservation planning, population assessment, and restoration (Table 1). Initially, the MUs were grouped based on LPCH dependency on each of the units (Table 1). Group A MUs are considered suitable habitat and are based on MUs where shin-oak or sand sagebrush are dominant, with minor to no honey mesquite. Areas in Group B are considered to be seasonal-use to transitional areas and consist of MUs dominated by native mid- or tall-grasses or grasslands with minor shin-oak components. Group C areas contain altered vegetation types that were originally LPCH habitat and areas that have been impacted by invasive species but still contain enough suitable vegetation for restoration. All other MUs are considered unsuitable habitat. Probable Conservation Reserve Program (CRP) lands are classified as unsuitable because, although LPCH are reported to use CRP areas in Kansas [34], we lack information regarding which CRP species and communities are used by LPCH and how they are used, particularly in New Mexico. CRP types are, however, identified on the map. When more data on LPCH use of CRP become available for New Mexico, parts of this MU could be included as potentially suitable habitat.

To identify areas of suitable habitat, we performed a patch size analysis including patches of 3,200 and 7,200 ha (2 and 3 mi radius, respectively). We included only Group A MUs as suitable habitat to avoid marginal habitats. An additional analysis identified patches ≥ 3,200 ha of the highest-quality habitats, MUs 1–3 (Table 1).

The map revealed large, regularly shaped patches of uniform mid- and tall-grasses, surrounded by native shinnery communities. We determined that these grasslands were converted from shin-oak-dominated communities to native grasslands. To identify and classify landscape changes, we developed an NDVI from a June 2002 image. A strong correlation has been demonstrated between NDVI and the phenology of land cover types, including desert shrublands [35], and NDVI has been used to quantify land use change individually or together with other indices and transformations [36,37], as well as to detect inter-annual variation in vegetation growth and senescence [38]. Our NDVI vividly discriminated between areas dominated by live, green vegetation, as opposed to tan or brown, senescent vegetation. To identify herbicide-treated areas, we used the NDVI image in conjunction with BLM Roswell, NM Field Office environmental assessment decision documents indicating the boundaries of areas treated with herbicide from 1981 to 1993. BLM last treated with tebuthiuron in 1993, and data are not available for treatment on private land. Areas of agricultural conversion were distinguishable primarily by circular patterns created by center-pivot irrigation. Where we were unable to identify a cause of disturbance, we classified the patch as unknown disturbance. For areas where we could positively identify agricultural conversion in the field and on the imagery, we used on-screen digitizing to correct the final map.

#### PPA analysis

At the request of the working group, we performed a separate analysis of occupied, suitable, potentially suitable, and unsuitable LPCH habitat within the PPA. The PPA is a 424,522 ha area defined by the working group in N Lea, S Roosevelt, and NE Chaves Counties. It contains the highest concentrations of LPCH in NM. A primary purpose of the PPA analysis was to provide a basis for strategic planning for oil and gas development. For this analysis, occupied habitat was defined by the working group as all areas within 1.5 miles of an active LPCH lek site, regardless of vegetation. The working group definition of a lek is two or more males that have been seen displaying during mating season at least one year out of the last five. Lek survey data were provided by BLM Roswell and Carlsbad Field Offices, NM Department of Game and Fish, and the Natural Heritage NM NMBiotics database.

For the PPA analysis, suitable habitat was considered to be unoccupied areas of appropriate vegetation type, in patches of 129.5 ha or more, falling entirely outside of avoidance distances around roads and oil and gas infrastructure. Avoidance distances are radii around infrastructure that LPCH avoid [25]. The 129.5 ha patch size (320 ac) was chosen by the working group to include patches currently too small to provide suitable habitat but large enough to provide restoration potential by filling in habitat gaps. For this analysis, appropriate vegetation types included: Shin-Oak/Mixed Mid-Grass & Tall-Grass Duneland (MU1), Shin-Oak/Sparse Duneland (MU2), Shin-Oak/Mixed Mid-Grass & Tall-Grass Shrubland (MU3), Shin-Oak/Mixed Mid-Grass & Short Grass Shrubland (MU5), Shin-Oak/Sparse Shrubland (MU6), Shin-Oak-Sand Sagebrush Shrubland (MU15), Sand Sagebrush Shrubland (MU8), Mixed Grasses/Shin-Oak Grassland (MU24), Mid-Grass Grassland (MU23), Tall-Grass Grassland (MU13), and CRP Fields (MU19).

Potentially suitable habitat was defined as unoccupied areas of appropriate vegetation type (see above list for suitable habitat), but in patches of less than 129.5 ha and/or falling within impact/avoidance distances [25] around oil/gas and road infrastructure. Unsuitable habitat was defined as areas outside of appropriate vegetation, including urban and agricultural areas, areas where shin-oak is naturally not present or has been eliminated by chemical treatment, or other areas where natural vegetation has been greatly altered or degraded.

## Abbreviations

BLM – Bureau of Land Management

CRP – Conservation Reserve Program

DOQ – digital ortho-photo quadrangle

ETM^+ ^– Enhanced Thematic Mapper^+^

GIS – geographic information system

LPCH – lesser prairie-chicken

MU – map unit

NDVI – normalized differential vegetation index

PPA – primary population area

SDL – sand dune lizard

## Authors' contributions

KJ participated in the stakeholder group, helped design analyses, supervised the study, and drafted the manuscript. TBN performed GIS analyses and assisted with manuscript preparation. PN performed image analysis for the map, consulted on GIS analyses, and assisted with manuscript preparation.
